# Postoperative occlusion of visual axis with fibrous membrane in the presence of anterior capsular phimosis in a patient with pseudoexfoliation syndrome: a case report

**DOI:** 10.1186/s12886-016-0394-y

**Published:** 2016-12-07

**Authors:** Eung Suk Kim, Moosang Kim, Seung-Jun Lee, Sang Beom Han, Hee Kyung Yang, Joon Young Hyon

**Affiliations:** 1 Department of Ophthalmology, Kyung Hee University Hospital, Kyung Hee University, Seoul, South Korea; 2 Department of Ophthalmology, Kangwon National University Hospital, Kangwon National University Graduate School of Medicine, 156 Baengnyeong-ro, Chuncheon, Kangwon 200-722 South Korea; 3 Department of Ophthalmology, Seoul National University Bundang Hospital, Seoul National University College of Medicine, Seongnam, South Korea

**Keywords:** Anterior capsular phimosis, Anterior segment optical coherence tomography, Case report, Fibrous membrane, Nd:YAG Laser, Pseudoexfoliation syndrome

## Abstract

**Background:**

To report a case of postoperative fibrous membrane formation occluding the visual axis in the presence of anterior capsular phimosis in a patient with pseudoexfoliation syndrome.

**Case presentation:**

A 79-year-old Asian woman with pseudoexfoliation syndrome underwent uneventful phacoemulsification and implantation of one-piece hydrophilic acrylic square-edged intraocular lens (Cristalens) in the right eye. Two months later, she had blurred vision in the right eye with the best-corrected visual acuity (BCVA) of 20/40. Formation of fibrous membrane occluding the capsulorhexis opening with contraction of anterior capsule was observed, which was confirmed by anterior segment optical coherence tomography. Clear visual axis was achieved by lysis of the membrane using Nd:YAG laser. The BCVA improved to 20/20.

**Conclusions:**

Occlusion of the visual axis with fibrous membrane can develop in the presence of anterior capsular phimosis in a patient with pseudoexfoliation syndrome.

## Background

Anterior capsular phimosis (or anterior capsular contraction syndrome) is one of the complications of cataract surgery that occurs associated with continuous curvilinear capsulorhexis (CCC) [1]. It was described as an exaggerated fibrotic response reducing the size of the anterior capsulorhexis opening and equatorial capsular diameter [1]. It typically occurs in patients with conditions of zonular weakness and intraocular inflammation [1, 2], such as pseudoexfoliation [3–5], uveitis [1, 2], myotonic dystrophy [2], and retinitis pigmentosa [6].

Previous reports showed that severe anterior capsular phimosis can cause complete occlusion of the pupil [2, 3, 7, 8]. However, through a comprehensive search of the MEDLINE database, occlusion of visual axis with fibrous membrane in the presence of incomplete anterior capsular contraction with capsulorhexis opening of 3.0 mm has rarely been reported [2]. Although anterior segment imaging modalities including anterior segment optical coherence tomography (AS-OCT) is expected to visualize the occlusion of the capsular opening, there has been no report of application of the AS-OCT in the condition.

We recently experienced a case of fibrous membrane formation occluding the visual axis accompanied with anterior capsular phimosis after an uneventful cataract surgery in a patient with pseudoexfoliation syndrome which was confirmed with AS-OCT, thus herein report the case.

## Case presentation

A 79-year-old Asian woman with pseudoexfoliation syndrome was referred for cataract surgery in the right eye. Her past medical history was unremarkable. Her best-corrected visual acuity (BCVA) was 20/50 in the right eye.

She underwent right phacoemulsification under topical anesthesia. As she had a small pupil of approximately 5.5 mm diameter, CCC with a diameter of 5.0 mm was performed without iris manipulation. Phacoemulsification was completed without any intraoperative complication, and a foldable one-piece hydrophilic acrylic square-edged intraocular lens (IOL) (22.0 diopters, 6.0mm optical diameter, 10.75mm overall diameter, model number: CLARE^®^; Cristalens, Paris, France) was implanted through a temporal corneal incision. Postoperatively, anti-inflammatory treatment with topical prednisolone acetate 1.0% 4 times daily was applied for 1 month. The BCVA was 20/40 at 1 week, which improved to 20/25 at 1 month.

**Fig. 1:**
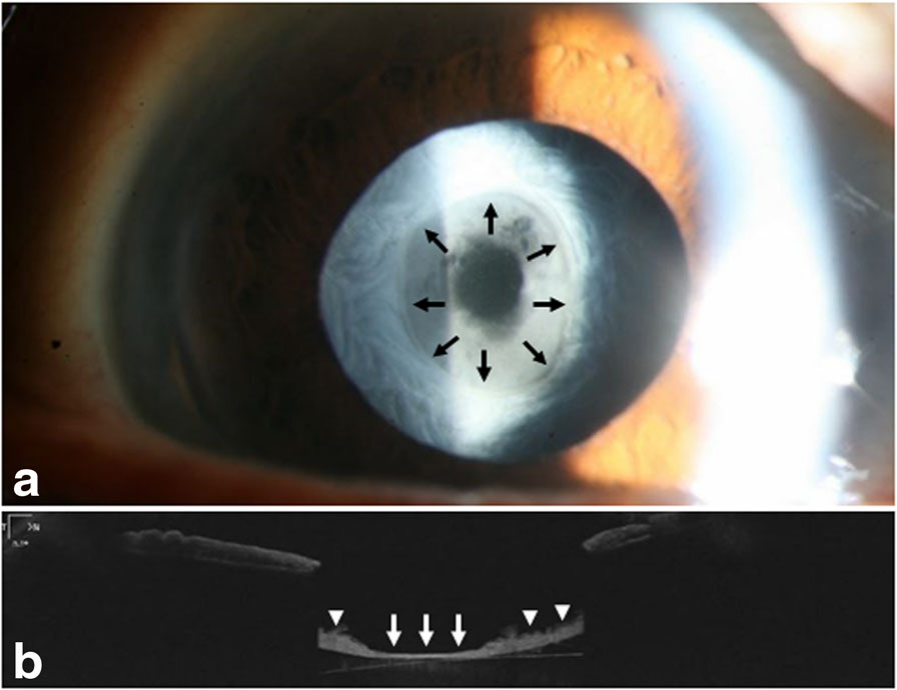
Anterior segment examnation of the right eye shows shrinkage of the anterior capsulorhexis opening and fibrous membrane formation occluding the opening. Margin of the anterior capsulorhexis is clearly visible (*black arrow*) (**a**). An AS-OCT image shows the presence of fibrous membrane occluding the visual axis (*white arrow*) and thick anterior capsule (*white arrowhead*) (**b**)

Two months after the surgery, she presented with blurred vision in the right eye. The BCVA was 20/40. Slit lamp examination after dilation revealed marked opacity and thickening of the anterior capsule. Anterior capsulorhexis opening reduced to a diameter of approximately 3.0 mm due to capsular contraction, and fibrous membrane occluding the capsulorhexis opening was observed (Fig. 1a). AS-OCT (Visante; Carl Zeiss Meditec, Oberkochen, Germany) confirmed the presence of the membrane (Fig. 1b). Slight anterior chamber (AC) cell reaction was found.

**Fig. 2:**
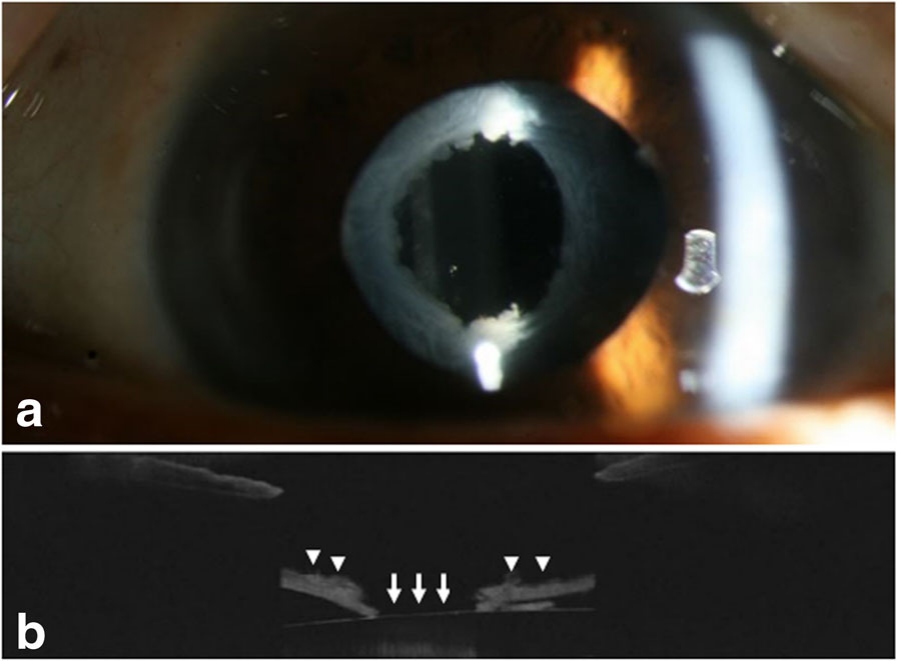
Immediately after the Nd:YAG laser treatment, anterior segment exmination of the right eye demonsrtates a clear anterior capsular opening (**a**). An AS-OCT image confirms removal of the fibrous membrane (*white arrow*) surrounded by thick anterior capsule (*white arrowhead*) (**b**)

A neodymium: yttrium aluminium garnet (Nd:YAG) laser was used to clear the visual axis. The membrane was lysed with a total of 24 mJ (15 shots × 1.6mJ) Nd:YAG laser. As a clear anterior capsular opening with a diameter of 3.0 mm was attained (Fig. 2a and b), no further excision of the anterior capsule was done. Topical prednisolone acetate 1.0% 6 times daily for 1 month was prescribed to prevent intraocular inflammation.

**Fig. 3:**
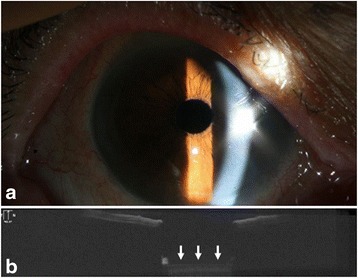
At 1 month after the Nd:YAG laser treatment, anterior segment examination of the right eye reveals clear visual axis (**a**). An AS-OCT image shows clear anterior surface of the IOL (*white arrow*) (**b**)

One month later, her BCVA improved to 20/20 in the right eye. Slit lamp examination revealed clear visual axis (Fig. 3a). AS-OCT demonstrated no membrane or pit on the anterior surface of the IOL (Fig. 3b).

## Discussion

In this case, AS-OCT was used to visualize the anterior capsular phimosis and formation of the membrane occluding the capsular opening.

In the present case, the occluding membrane as well as the anterior capsular phimosis was detected at 2 months postoperatively, which is in agreement with the previous reports that maximal rate of capsular contraction occurred within postoperative 6 weeks [7, 9]. Clear visual axis was observed at one month after the Nd:YAG laser treatment. As capsular stability is reported to be achieved at 3 months postoperatively [10], we expect that the capsulorhexis can be stabilized. However, we also believe further follow-up is needed as there is possibility of further capsular contraction, particularly because the patient has pseudoexfoliation syndrome.

Anterior capsular phimosis is postulated to consist of two mechanisms: 1) capsular shrinkage, probably due to actin filaments within residual lens epithelial cells (LECs) and 2) proliferation and fibrous metaplasia of these residual LECs which lead to the reduction of the size of the capsulorhexis opening [2, 7]. Histopathological examination showed that the proliferative membrane was composed of subcapsular fibrous tissue interspersed with proliferated fibrocytic cells, derived from residual LECs [2, 7]. Using scanning electron microscope, Ueno et al. [11] demonstrated the presence of fibroblast-like cells in the area of the anterior capsular occlusion. Kurosawa et al. [12] also revealed that anterior capsular phimosis involved outgrowth of fibrous tissue from the capsule margin and its contraction.

To our knowledge, anterior capsular phimosis after implantation of the Cristalens CLARE IOL has never been reported. Although hydrophilic acrylic IOLs with square-edge design and four haptics are expected to have enhanced uveal biocompatibility and capsular support [3, 13, 14], a few cases of anterior capsular phimosis after implantation of these IOLs were reported [4, 5, 15]. Notably, most of the cases were associated with pseudoexfoliation syndrome [4, 5]. There have been several case reports of anterior capsular phimosis in patients with pseudoexfoliation syndrome despite the insertion of capsular tension ring [3, 5, 16]. Pseudoexfoliation syndrome appears to significantly increase the risk of anterior capsular phimosis due to the following reasons: 1) As capsular shrinkage is conceivably associated with an imbalance between centripetal and centrifugal forces that act on the zonules and the capsulorhexis edge [3], zonular weakness can exaggerate the contraction response. 2) Although larger CCC is correlated with less capsule contraction [17], small CCC is often inevitable in pseudoexfoliation syndrome due to poor mydriasis. 3) Complete cleansing of LECs is also important for the prevention of the fibrous proliferation [17]. However, thorough removal of the LECs, particularly those at the lens equator, is often difficult due to small pupil. 4) A compromised blood-aqueous barrier in the condition may result in increased postoperative inflammation, which can precipitate the progression of anterior capsular phimosis [1, 5].

In the present case, fibrous membrane occluding the anterior capsulorhexis opening developed in the presence of capsular phimosis with capsulorhexis opening of 3.0 mm. We postulate that the phenomenon was due to the following mechanisms: 1) Formation of the fibrous membrane could be faster than the progression of the capsular contraction, which might cause the occluding membrane formation before marked reduction of the capsular opening size. 2) The design (square-edged one piece with 4 haptics) and material (hydrophilic acrylic) of the IOL might exert high strength of capsular support, which could help maintain the capsular opening despite the fibrous proliferation. Spang et al. [2] reported a similar case of anterior capsular phimosis in which proliferated LECs filled the capsular opening. In their case, they used an IOL with 13.5mm overall length, which might be advantageous for capsular support [2]. Another remarkable thing is that we used substantially less Nd:YAG laser energy compared to laser energy of 90 to 140 mJ used in other reports [3, 5], probably because only removal of the fibrous membrane without manipulation of the capsule was needed to clear the visual axis.

## Conclusions

We report a case of membrane formation occluding the visual axis in the presence of anterior capsular phimosis after implantation of Cristalens CLARE IOL in a patient with pseudoexfoliation syndrome. Nd:YAG laser can be effective in the treatment of the condition.

## Abbreviations

AC: Anterior chamber
AS-OCT: Anterior segment optical coherence tomography
BCVA: Best-corrected visual acuity
CCC: Continuous curvilinear capsulorhexis
IOL: Intraocular lens
LECs: Lens epithelial cells
Nd:YAG: Neodymium: yttrium aluminium garnet

## References

[1] Davison JA (1993). Capsule contraction syndrome. J Cataract Refract Surg.

[2] Spang KM, Rohrbach JM, Weidle EG (1999). Complete occlusion of the anterior capsular opening after intact capsulorhexis: clinicopathologic correlation. Am J Ophthalmol.

[3] Moreno-Montanes J, Sanchez-Tocino H, Rodriguez-Conde R (2002). Complete anterior capsule contraction after phacoemulsification with acrylic intraocular lens and endocapsular ring implantation. J Cataract Refract Surg.

[4] Zaugg B, Werner L, Neuhann T (2010). Clinicopathologic correlation of capsulorhexis phimosis with anterior flexing of single-piece hydrophilic acrylic intraocular lens haptics. J Cataract Refract Surg.

[5] Dubois VD, Ainsworth G, Liu CS (2009). Unilateral capsular phimosis with an acrylic IOL and two capsular tension rings in pseudoexfoliation. Clin Experiment Ophthalmol.

[6] Sudhir RR, Rao SK (2001). Capsulorhexis phimosis in retinitis pigmentosa despite capsular tension ring implantation. J Cataract Refract Surg.

[7] Edrich CL, Ghanchi F, Calvert R (2005). Anterior capsular phimosis with complete occlusion of the capsulorhexis opening. Eye (Lond).

[8] Kim EC, Hwang HS, Kim MS (2013). Anterior capsular phimosis occluding the capsulorhexis opening after cataract surgery in a diabetic patient with high hemoglobin A1C. Semin Ophthalmol.

[9] Walsh LM, Pande M (1998). Capsulorhexis phymosis following uncomplicated phacoemulsification surgery. Eye (Lond).

[10] Hayashi K, Hayashi H, Nakao F, Hayashi F (1997). Reduction in the area of the anterior capsule opening after polymethylmethacrylate, silicone, and soft acrylic intraocular lens implantation. Am J Ophthalmol.

[11] Ueno S, Kimura A, Hirata A, Tanihara H (2004). Surgical treatment of complete anterior capsule contraction after cataract surgery. Acta Ophthalmol Scand.

[12] Kurosaka D, Ando I, Kato K (1999). Fibrous membrane formation at the capsular margin in capsule contraction syndrome. J Cataract Refract Surg.

[13] Abela-Formanek C, Amon M, Schauersberger J, Kruger A, Nepp J, Schild G (2002). Results of hydrophilic acrylic, hydrophobic acrylic, and silicone intraocular lenses in uveitic eyes with cataract: comparison to a control group. J Cataract Refract Surg.

[14] Werner L, Pandey SK, Apple DJ, Escobar-Gomez M, McLendon L, Macky TA (2001). Anterior capsule opacification: correlation of pathologic findings with clinical sequelae. Ophthalmology.

[15] Cavallini GM, Masini C, Campi L, Pelloni S (2008). Capsulorhexis phimosis after bimanual microphacoemulsification and in-the-bag implantation of the Akreos MI60 intraocular lens. J Cataract Refract Surg.

[16] Kurz S, Krummenauer F, Hacker P, Pfeiffer N, Dick HB (2005). Capsular bag shrinkage after implantation of a capsular bending or capsular tension ring. J Cataract Refract Surg.

[17] Joo CK, Shin JA, Kim JH (1996). Capsular opening contraction after continuous curvilinear capsulorhexis and intraocular lens implantation. J Cataract Refract Surg.

